# Motor relearning program along with electrical stimulation for improving upper limb function in stroke patients: A quasi experimental study

**DOI:** 10.12669/pjms.36.7.2351

**Published:** 2020

**Authors:** Ikram Ullah, Aatik Arsh, Aneela Zahir, Shafqatullah Jan

**Affiliations:** 1 Ikram Ullah, BSPT, PP-DPT, MSPT. Physiotherapist, Saidu Group of Teaching Hospitals Swat, Pakistan; 2 Aatik Arsh, DPT, MSPT. Lecturer, Institute of Physical Medicine and Rehabilitation, Khyber Medical University Peshawar, Pakistan; 3 Aneel Zahir, DPT. Lecturer, Department of Health Sciences, NCS University System Peshawar, Pakistan; 4 Shafqatullah Jan, BSPT, MSPT. Physiotherapist, Pakistan Institute of Prosthetics and Orthotic Sciences Peshawar, Peshawar, Pakista

**Keywords:** Cerebrovascular accident, Electrical stimulation, Physical Therapy, Rehabilitation, Stroke

## Abstract

**Objective:**

To determine the effectiveness of motor relearning program along with electrical stimulation for improving upper limb function in patients with sub-acute stroke.

**Methods:**

A quasi experimental study was conducted at Physiotherapy Department of SAIDU Group of Teaching Hospitals Swat Khyber Pakhtunkhwa from January to June 2019. Forty four subjects with post stroke duration of 3-9 months (sub-acute) participated in the study. Subjects received electrical stimulations for the effected arm for 15 minutes along with motor relearning programme for an hour five days a week for six weeks. The upper limb sub scales of motor assessment scale were used to collect pre and post treatment data. SPSS version 20 was used to analyze the data.

**Results:**

The mean age of the participants was 54.95±13.2 years. Out of 44 participants 31(70.5%) were male and 13 (29.5%) were female. Pretreatment upper arm function, hand movement and advance hand activities scores were 1.36 ± 0.49, 1.18 ± 0.39 and 1.04 ± 0.21 respectively while their post treatment scores were 5.18 ± 0.96, 4.77 ± 1.02 and 3.95 ± 1.21 respectively. There was significant differences (P<0.05) between pre and post treatment scores of upper arm function, hand movement and advance hand activities.

**Conclusion:**

Motor relearning program along with electrical stimulation significantly improves upper limb function in patients with sub-acute stroke.

## INTRODUCTION

Following coronary heart disease and cancer, stroke (cerebrovascular accident) is the third most common cause of death throughout the world.^1^ About 5.5 million people died because of stroke, and about 20% of these deaths occur in South Asia. The exact burden of stroke in Pakistan is unknown, however, according to conservative estimates the annual incidence of stroke in Pakistan is 250/100,000.^2^,^3^ Stroke is one of the leading causes of physical disability in adult population. The most disabling condition that confines independent life of stroke patients is the upper limb motor impairment. About 85% patients has motor impairments in affected upper limb with first episode of stroke and this upper extremity motor deficit remain in 55 to75% patients even after six months of stroke. Full recovery of arm weakness occurs only in 5-20% of patients.^4^,^5^ Due to weakness of arm, stroke patients have difficulty in reaching, grasping and manipulation of objects, which causes difficulty in performing activities of daily living. Moreover, with paretic limb, these patients cannot perform simple tasks such as grooming, eating meal, dressing and undressing of cloth. Majority of stroke patients consider upper limb weakness the major problem and this is related with a decrease level of subjective well-being.^4^-^6^

In clinical settings, a number of interventions ranging from simple therapeutic exercises to sophisticated electromechanically devices are applied to improve upper limb function in stroke patients. Literature support the use of proprioceptive neuromuscular facilitation, constrained induced movement therapy, mirror therapy and the most recently developed motor relearning programme (MRP).^7^ MRP was proposed by Carr and Shepherd with the assumption that motor relearning requires repetitive task specific training. Quite a few research studies reported that MRP enhances restoration of upper extremity function.^8^,^9^

Besides these specific therapeutic approaches, varieties of physical therapy modalities are used for the rehabilitation of stroke patient. Electrical stimulation is one of the most commonly used electrotherapeutic modality in clinical practice which is used to stimulate the paretic limb.^10^,^11^ Literature suggest that electrical stimulation can be used in stroke patients, however, electrical stimulation results in passive movements, that’s why functional training cannot be performed with electrical stimulation.^10^-^12^ To overcome this discrepancy, task-oriented repetitive exercises in the form of MRP may be applied along with electrical stimulation to improve functional movements. There is limited literature available regarding the use of electrical stimulation along with MRP, therefore current study was designed to determine the effectiveness of motor relearning program along with electrical stimulation for improving upper limb function in patients with sub-acute stroke.

## METHODS

A quasi experimental study was conducted at physiotherapy department of Saidu Group of Teaching Hospitals Swat Khyber Pakhtunkhwa from January to June 2019. Permission was taken from administration of Saidu group of teaching hospitals Swat before the commencement of the study. Ethical approval was obtained from ethics board of Khyber Medical University Peshawar (DIR/KMU-EB/EM/000439).

Stroke patients with following inclusion criteria were included in the study: 1. Male and female patients of age 30-65 years with first episode of unilateral stroke-2. Patients with post stroke duration of 3-9 months 3. Cognitively stable patients with Mini mental state examination scores 24 or more. Those patients were excluded who had pain of score three or more on numeric pain rating scale in the affected limb or who had any other major medical complication.

Forty-four subjects were recruited for the the study through convenience sampling. Informed consent was obtained from all patients and their caregivers. All the subjects were assessed by a trained physical therapist and information about the study interventions were provided to each patient. The interventions consisted of motor relearning program and electrical stimulation, which were provided for five days a week for six weeks. There was one session per day and each session was of one hour. In each session, motor relearning program was applied for 45 minutes while electrical stimulation was applied for 15 minutes.

Motor relearning program comprised of following functional activities: opening/closing lid of bottles, picking the water in glass and drink it, arranging puzzles, reach and manipulate the glass of water in different directions and putting into the mouth, pick small objects from one container to another, turning doors handgrips, reading magazine and turning the pages of books or newspaper. The exercises regime was designed according to the motor deficit of the individual patient. If the task or function was difficult for the patient to perform, then those tasks were fragmented into different parts so that the patient can easily perform it. Generally, each exercise or task was repeated 10 to 15 time with affected arm. The progressive increase in tasks was so adopted that as the patient in study improved, the task became difficult and complicated. For example to improve grasping, initially large tennis ball was used and as the patient improved smaller ball was used to train the patient in grasping and relearning of every day routine activities.

The electrical stimulation was applied in the form of faradic current to the affected arm at the rate of 90 visible contractions per motor point on the affected muscles through surface electrodes. The patient was supine lying or in sitting position so that he could see his muscle contractions. No pain was elicited during the whole stimulation period; if the patient pain threshold was low then intensity of the current was decreased accordingly.

The upper limb sub scales (1-upper arm function, 2-hand movement, 3-advance hand activities) of motor assessment scale were used to collect pre and post treatment data. SPSS version 20 was used to analyze the data. Shapiro-wilk test was applied to check normality of data. Because data was normally distributed that’s why parametric test (paired sample T-test) was used to compare pre and post treatment data.

## RESULTS

The mean age of the participants was 54.95 ± 13.2 years. Out of 44 participants, 31(70.5%) were male and 13 (29.5%) were female. Majority (n=36, 72.7%) of the participants had ischemic stroke while remaining 12 (27.3%) participants had hemorrhagic stroke. About half (n=23, 52.3%) of the patients had left hemiplgia while 21(47.7%) had right hemiplgia.

Pretreatment upper arm function, hand movement and advance hand activities scores were 1.36 ± 0.49, 1.18 ± 0.39 and 1.04 ± 0.21 respectively while their post treatment scores were 5.18 ± 0.96, 4.77 ± 1.02 and 3.95 ± 1.21 respectively. There was significant differences (P<0.05) between pre and post treatment scores of upper arm function, hand movement and advance hand activities. (Table-I)

**Table-I:** Pre and post treatment scores of Upper arm function, Hand movement and Advance hand activities.

Outcome-measures	Pre-treatment Mean ± S.D	Post-treatment Mean ± S.D	Mean difference	P-value
Upper arm function	1.36 ± 0.49	5.18 ± 0.96	3.82	0.001
Hand function	1.18 ± 0.39	4.77 ± 1.02	3.59	0.001
Advance hand activities	1.04 ± 0.21	3.95 ± 1.21	2.91	0.001

## DISCUSSION

Upper limb impairments are common in stroke patients and rehabilitation specialists always give more focus to upper extremity movements because they are necessary for performing almost all activities of daily living.^13^,^14^ Without good upper limb function, stroke patients remain dependent for the rest of their lives and they never ever function as productive members of the society.^15^ Different rehabilitation approaches and physical therapy modalities can be used to restore upper limb function in stroke patients.^16^ Despite the fact that MRP and electrotherapy are commonly used by rehabilitation specialists in clinical settings, however few studies evaluated combined effects of these two interventions. Current study evaluated the effects of MRP along with electrotherapy in the management of upper extremity functions in stroke patients.

In current study, only sub-acute patients with stroke duration of three to nine months were included. Previous studies reported that due to natural recovery, maximum restoration of upper limb function in stroke patients occurs in first three months post stroke. However, after three months, stroke patients mostly remain in stable condition.^13^,^17^,^18^ To avoid confounding effect of spontaneous recovery, acute stroke patients were not included in current study. Contrast to acute cases, chronic stroke patients often develops secondary complications including contractures, synergies and chronic pain syndromes.^19^,^20^ These secondary complications may limit the effectiveness of the interventions, that’s why chronic stroke patients were excluded from current study.

The results of current study reported that MRP along with electrical stimulation significantly improves upper limb function in stroke patients. Though current study reported effectiveness of these interventions only for sub-acute stroke patients, however, they may be used in other stroke patients. The results of current study are in agreement with the study of Hsu et al. which reported significant improvement in motor function of upper limb in hemiplegic patients after intervention of electrical stimulation for four weeks, along with regular indoor rehabilitation exercises.^12^ Chae et al. also reported that different types of electrical stimulations in the rehabilitation of stroke patients significantly improves motor function in stroke patients.^21^

The type of electrical stimulation used in this study was of surface-electrode electrical stimulation. Literature shows, that this type of electrical stimulations is useful in improving motor impairment in stroke patients, however literature regarding its effects on functional outcomes is scarce.^22^-^24^ Results of current study showed that electrical stimulation along with MRP significantly improves upper limb functions. De Kroon et al. reported positive effects of surface-electrode electrical stimulation on motor relearning.^25^ Literature suggest that electromyography-triggered electrical stimulation may be more useful than surface stimulations.^26^ But as compared to sophisticated electromyography-triggered electrical stimulation, surface-electrode electrical stimulation is cost-effective and can be administered easily. It is worthy to mention that Hummelsheim et al. conducted a study in which they compared electrical stimulation with repetitive hand and task oriented exercises in stroke patients. The results of their study showed no significant improvement upper limb function after two weeks of electrical stimulation but found improvement in upper limb function after two weeks of motor relearning programme.^27^ To sum up, it can be concluded on the basis of available literature that electrical stimulation can improve motor impairments of upper limb but it may not have significant effects on upper limb function. Nevertheless, electrical stimulation combined with MRP may significantly improve upper limb function in stroke patients. There is scarce literature regarding the use of MRP along with electrical stimulation in the management of upper limb function, however, the previously conducted studies shows promising results.

### Limitation of the study

Despite the fact that current study was one of the preliminary study, which reported effectiveness of MRP along with electrical stimulation for improving upper limb function in stroke patients, however, it has some limitations. Current study was a quasi-experimental study and due to lack of control group, it was not possible to compare outcomes between groups. Secondly, current study was conducted in clinical settings, so confounding variables were difficult to control. Moreover, long term effects of the intervention were not evaluated.

## CONCLUSION

MRP along with electrical stimulation significantly improves upper limb function in patients with sub-acute stroke. Large clinical trials and multicenter studies are recommended to truly determine effectiveness of MRP and electrical stimulation in the management of upper limb impairments in stroke patients.

### Author’s Contribution:

**IU:** Concept and study design, literature search and literature review, Acquisition of data, drafting the manuscript, responsible and accountable for the accuracy and integrity of the work.

**AA:** Concept and study design, data analysis and interpretation, drafting the manuscript, Final approval of the version to be published.

**AZ:** Literature search and literature review, data analysis and interpretation, Critical revision.

**SJ:** Acquisition of data, Data analysis and interpretation, critical revision.
